# Supplementary material for: Homeostatic control of deep sleep and molecular correlates of sleep pressure in Drosophila
Source: eLife. 2023 Oct 31;12:e91355. doi: 10.7554/eLife.91355 (PMC10642965; doi:10.7554/eLife.91355)
Supplement: Supplementary file 1. [file elife-91355-supp1.docx]

**SUPPLEMENTARY ONLINE MATERIAL**

**Homeostatic Control of Deep Sleep and Molecular Correlates of Sleep Pressure in *Drosophila***

Budhaditya Chowdhury^1^*, Lakshman Abhilash^1^*, Antonio Ortega^2^, Sha Liu^2^, Orie Shafer^1#^

^1^ The Advanced Science Research Center, The City University of New York; The Graduate Center at the City University of New York

^2^ VIB-KU Leuven Center for Brain & Disease Research, Leuven, Belgium

*These authors contributed equally

*Running title: Drosophila sleep homeostasis*

^#^Correspondence: [oshafer@gc.cuny.edu](mailto:oshafer@gc.cuny.edu)

**Figure 1-figure supplement 1:** Time courses of “active sleep” – bouts of inactivity that are between one to five minutes long – under conditions of 6 **(A)**, 12 **(B)** and 24-h **(C)** sleep deprivation using vortexers at a trigger frequency of 220-s for *CS* flies. Plots are means±SEM. Gray shaded regions indicate the dark phase of the LD cycle. Red shaded regions along the *x*-axis indicate windows of sleep deprivation.

**Figure 2-figure supplement 1. A.** Activity time course of undisturbed and sleep-deprived flies across the 24 hours following mechanical deprivation. During ZT12-24 on post SD day 1, focal flies display elevated night-time activity at times corresponding to the normal period of consolidated sleep. **B**. The activity of focal flies between ZT12-24 was significantly higher than that of undisturbed controls (Wilcoxon’s *W* = 2902, *p* = 0.009). **C.** Activity time course of focal and yoked flies during the same Post deprivation window (24-H). **D.** Activity of focal and yoked flies during ZT12-24 were not significantly different from each other (Wilcoxon’s *W* = 2902, *p* = 0.06). *n* = 82 each for focal and yoked categories, and *n* = 58 for unperturbed controls.

**Figure 3-figure supplement 1. A.** Time course of conditional Doze probability – p(Doze) – for focal and yoked *CS* controlled flies across three cycles post sleep deprivation using 220-s trigger frequency. Plotted are means±SEM. **B.** Changes in p(Doze) values compared to baseline. Plotted are means±SEM. Statistically significant differences between focal and yoked flies were inferred using a two-sample, one tailed t-tests for unequal variances (Post1: Day – *t_161.99_* = 2.64, *p* = 0.005; Night – *t_159.8_* = 1.56, *p* = 0.06; Post2: Day – *t_157.66_* = 0.95, *p* = 0.17; Night – *t_159.83_* = 2.2, *p* = 0.01; Post3: Day – *t_161.41_* = 0.64, *p* = 0.26; Night – *t_161.93_* = 2.7, *p* = 0.004). Gray shaded regions indicate dark phase of the LD cycle, in both panels. * p < 0.05, ** p < 0.01, NS – Not Significant.

**Figure 3-figure supplement 2. A.** Comparison of bout numbers for standard sleep between focal and yoked flies in three post-deprivation cycles did not reveal significant differences (Post1: Wilcoxon’s *W* = 3385, *p* = 0.94; Post 2: Wilcoxon’s *W* = 2962.5, *p* = 0.18; Post 3: Wilcoxon’s *W* = 3273, *p* = 0.77). **B.** Comparing bout duration of standard sleep across three post deprivation cycles revealed that focal flies had significantly higher bout durations for the first two recovery cycles (Post1: Wilcoxon’s *W* = 4077, *p* = 0.01; Post 2: Wilcoxon’s *W* = 4269, *p* = 0.002; Post 3: Wilcoxon’s *W* = 3624, *p* = 0.38). *n* = 82 each for focal and yoked categories. Undisturbed control n = 58 * < 0.05, and N.S. (Not Significant). **C.** Pre-deprivation bout numbers (Wilcoxon’s *W* = 3358, *p* = 0.99) and bout durations (Wilcoxon’s *W* = 3705, *p* = 0.25) were not significantly different between focal and yoked pairs). *n* = 82 each for focal and yoked categories, and n = 58 for unperturbed controls * < 0.05, and N.S. (Not Significant).

**Figure 4-figure supplement 1. A.** frequency distribution of 25-min sleep bouts in undisturbed flies collected from 5 cycles. Every fly displayed at least one bout of sleep that was 25-min or longer. **B.** Total bout numbers in 25-min, long bout sleep across two cycles following 24-h of deprivation using 220-s inactivity triggers showed significant increases in focal flies compared to yoked flies (Post1: Wilcoxon’s *W* = 4029, *p* = 0.02; Post 2: Wilcoxon’s *W* = 4186.5, *p* = 0.006; Post 3: Wilcoxon’s *W* = 3779.5, *p* = 0.16). **C.** Duration of long bouts are not significantly different between focal and yoked flies in three post-deprivation days (Post1: Wilcoxon’s *W* = 3422, *p* = 0.53; Post 2: Wilcoxon’s *W* = 3229.5, *p* = 0.66; Post 3: Wilcoxon’s *W* = 3888, *p* = 0.08). *n* = 82 each for focal and yoked categories, and n = 58 for unperturbed controls * < 0.05, and N.S. (Not Significant).

**Figure 5-figure supplement 1.** Time course of control *CS* flies for five LD cycles from a different run (see and compare with Figure 4), showing the remarkable consistency between runs in the amount of 25-minute sleep. Plotted are means±SEM and dark shaded regions indicate the dark phase of the LD cycle.

**Figure 5-figure supplement 2. A.** Long-sleep bouts prior to deprivation were not different between focal and yoked flies (Wilcoxon’s *W* = 263, *p* = 0.09). **B.** On post-deprivation day 1 focal flies showed significantly increased long bout numbers (Wilcoxon’s *W* = 304, *p* = 0.004). On second day even though a trend was seen, it was not significant (Wilcoxon’s *W* = 244.5, *p* = 0.23), before returning to significant levels on post-deprivation day 3 (Wilcoxon’s *W* = 295, *p* = 0.009). **C.** Long bout duration on baseline days was not different between focal and yoked flies (Wilcoxon’s *W* = 225.5, *p* = 0.32). **D.** On three post derivation days, focal and yoked pairs did not show significant differences in long bout durations (Post1: Wilcoxon’s *W* = 210.5, *p* = 0.57; Post 2: Wilcoxon’s *W* = 231.5, *p* = 0.24; Post 3: Wilcoxon’s *W* = 242.5, *p* = 0.14). *n* = 20 each for focal and yoked categories. * < 0.05, and N.S. (Not Significant).

**Figure 6-figure supplement 1.** Spotted amines in MALDI-TOF MS experiments and detected serotonin concentration at m/z 177.07. Water was used as a solvent to make 1mg/ml concentrations for spotting slides.
